# Basin stability measure of different steady states in coupled oscillators

**DOI:** 10.1038/srep45909

**Published:** 2017-04-05

**Authors:** Sarbendu Rakshit, Bidesh K. Bera, Soumen Majhi, Chittaranjan Hens, Dibakar Ghosh

**Affiliations:** 1 Physics and Applied Mathematics Unit, Indian Statistical Institute, Kolkata-700108, India; 2 Department of Mathematics, Bar-Ilan University, Ramat Gan 52900, Israel

## Abstract

In this report, we investigate the stabilization of saddle fixed points in coupled oscillators where individual oscillators exhibit the saddle fixed points. The coupled oscillators may have two structurally different types of suppressed states, namely amplitude death and oscillation death. The stabilization of saddle equilibrium point refers to the amplitude death state where oscillations are ceased and all the oscillators converge to the single stable steady state via inverse pitchfork bifurcation. Due to multistability features of oscillation death states, linear stability theory fails to analyze the stability of such states analytically, so we quantify all the states by basin stability measurement which is an universal nonlocal nonlinear concept and it interplays with the volume of basins of attractions. We also observe multi-clustered oscillation death states in a random network and measure them using basin stability framework. To explore such phenomena we choose a network of coupled Duffing-Holmes and Lorenz oscillators which are interacting through mean-field coupling. We investigate how basin stability for different steady states depends on mean-field density and coupling strength. We also analytically derive stability conditions for different steady states and confirm by rigorous bifurcation analysis.

Different types of collective behavior emerge when two or more dynamical units interact with each other and suppression of oscillation is one of the most interesting phenomena among them. Oscillation quenched states are categorized in two processes named as amplitude death (AD)^1^ and oscillation death (OD)^2^. AD state is a result of stable homogeneous steady state (HSS), where all the oscillators merge or converge in one common steady state. In the case of OD state, oscillators populate to different stable steady states which are coupling dependent fixed points termed as stable inhomogeneous steady states (IHSS) and these states are the results of symmetry-breaking bifurcations in coupled oscillators. Also network of coupled oscillators exhibit multi-cluster oscillation death (MCOD) in nonlocally coupled oscillators^3^. MCOD pattern refers to the stabilization of various coupling dependent steady states to which the oscillators converge. Depending upon the initial conditions of each oscillator, the positions of the stable steady states for MCOD state may vary. AD state has a great importance to suppress unwanted oscillations. Such oscillations are responsible for obstructing certain process in some biological systems^4^ and laser experiments^5^. Due to ushering of inhomogeneity in homogeneous systems, OD state is very complicated phenomena and closely related to many biological processes such as cellular differentiation^6^, also in neural networks^7^ and synthetic genetic oscillators^8^,^9^. Recently, the transition from AD to OD state via Turing type bifurcations has been articulated^10^. Later many researchers have explored such transition using different types of coupling strategies such as mean-field^11^, presence of direct and indirect coupling^12^, mean repulsive interaction^13^. Also cyclic type of interaction^14^ can induce AD-OD transition in mismatched coupled systems. Beside IHSS (i.e. OD) state, there are many stable steady states which are also coupling dependent states known as non-trivial homogeneous steady states (NHSS). In ref. 15, the authors discussed about the suppression of mixed mode oscillations state in coupled oscillators. As AD state is a result of stabilization of HSS so it may be easy to derive the analytical condition for stability but in case of coupling dependent stable steady states (OD and NHSS), it is not always possible to obtain the stability condition analytically since OD states are multi-stable by nature. Most of the previous results on OD states are characterized by only bifurcation analysis and there is no clear discussion about the basin of multi stable OD states. So it is interesting to study the variations of such multi stable steady states with respect to the basins of attractions because multi stable steady states are omnipresent in many coupled dynamical systems.

Up to now, the stability of such collective steady states (AD or OD) in coupled network are characterized by the sign of real parts of eigenvalues of the corresponding Jacobian matrix. This linear stability analysis is valid only for infinitesimal perturbation near the steady states. So, the linear stability analysis is necessary for the stability of steady state but not sufficient against some significant perturbations. Since non-small perturbation is ubiquitous in nature and many man-made systems, so we need a global measure to characterize the stability. In this context a pioneer work^16^, they have developed a universal measure in complex systems as *basin stability* (BS) which is related to the volume of basin of attraction. The concept of BS has a lot of applications in real-world systems such as power grids^17^, arrays of coupled lasers^18^ etc. and effectively applied in many field of science^19^,^20^,^21^ that interplays with the systems which exhibit multi-stability. In practical situation such as human brain^22^,^23^, cell regulatory network^24^ and many other natural phenomena^25^,^26^,^27^ show the multi-stable behavior^28^ and also in the economics and social sciences^29^,^30^,^31^,^32^, the path dependence processes are suitably described by multistability. To quantify the stability of such multistable states in dynamical systems, the BS measure is successfully applied in finite^16^,^33^,^34^ as well as infinite dimensional systems^35^. The BS approach is well studied in various types of emergent and collective behavior in network of dynamical systems such as synchronizability^36^ of static and time varying complex network^37^ and many others but BS measure in quantification of different multi-stable steady states in coupled systems has not been explored yet, to the best of our knowledge. Therefore, systematic studies on such unnoticed phenomena deserve special attention.

In this work, we are dealing with finite dimensional systems and trying to give BS measure for oscillation suppression states (such as AD, OD, NHSS and MCOD) in a network of coupled dynamical systems. Oscillation cessations are significantly applied in many biological and physical processes where unwanted oscillations may arise so we need to suppress the oscillations to some desired stable steady states. We consider a network of globally and randomly connected oscillators through mean-field coupling. This mean field coupling is a natural coupling scheme which is extensively studied for different consequence in physics^38^, ecology^39^, biology^4^,^40^, chemistry, electrical circuits^1^,^2^. Also this type of interaction arises in metapopulation ecology where by proper tuning of mean-field density parameter, two-patch ecosystems are evolving from an open patchy ecosystems to closed patchy ecosystems^39^. The role of mean-field density is also discussed in ref. 8 and 40 in the context of intercell communication of synthetic gene oscillators via a small autoinducer molecule. In general, the mean-field coupling is applied in a network of dynamical systems where each oscillator is having equal chance of uniform interaction from all the oscillators. On the other hand, there are various types of stable steady states, which may not be possible to detect analytically from linear stability analysis due to their multistable behavior. AD state can never be produced in identical coupled systems using simple diffusive interaction but OD states may generate by proper choice of initial conditions and linear stability analysis fails to characterize such OD states due to multi stability. For such limitations, it is not possible to get any information about the stability of OD state against any non-small random perturbation from the state. Again, there exists Lyapunov function based approach^41^,^42^ as a process in determining the stability of different steady states locally as well as globally but unfortunately there is no systematic way to construct Lyapunov functions for high dimensional systems and it depends on the exact form of the governing system. So in order to do the present work we avoid such limitation and concentrating on this intriguing BS approach. Thus it is significant to quantify all the multi stable steady states by BS measure. The value of BS lies in [0, 1] and quantifies what amount stable a state is in probabilistic sense against the basin volume. With the help of this measurement all coupling dependent steady states (OD, NHSS) as well as coupling independent state (i.e. AD state) can be quantified. The effect of coupling strength on the variation of different stable states is quantified in BS framework. In BS measure, we integrate the whole network with a large population of initial states and give some probabilistic measure with respect to those initial points in the state space. We obtain analytical conditions of stabilization of various steady states that show excellent matching with our numerical simulations. Using rigorous bifurcation analysis we verify the results obtained analytically and appraise them by BS approach. For our investigation, we take coupled paradigmatic Duffing-Holmes and chaotic Lorenz oscillator to check the validation of our BS approach for global and random networks.

## Results

We start with a network of coupled oscillators with the isolated dynamics of each node of the network is given by 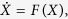 where *X* is a *m*-dimensional vector of the dynamical variables and *F*(*X*) is the vector field. The general framework of coupled network is given by the following equation:


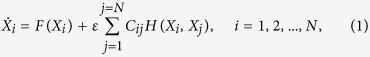


where *N* is the total number of nodes in the network, *ε* is the coupling strength, *C*_*ij*_ are the elements of connectivity matrix and *H*(*X*_*i*_, *X*_*j*_) is the coupling function between *i*-th and *j*-th node.

### Duffing-Holmes oscillator

We first consider a two-dimensional physical example, namely Duffing-Holmes (DH) oscillator^43^:


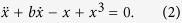


The oscillator has three steady states, namely two symmetrical stable steady states (±1, 0) (which are spiral or node depending on the damping coefficient *b* > 0) and a saddle point at (0, 0) irrespective of the values of the parameter *b*. For *b* < 0 each individual DH oscillator exhibit oscillatory state. Recently, Tamaševičiūtė *et al*.^44^ discussed the stabilization of saddle fixed points of an uncoupled DH oscillator using modified unstable filter^45^ method. The proposed technique is applicable only for *b* > 0 where the DH oscillator is either stable node or spiral. But they did not discuss the stabilization of saddle point in coupled oscillators. Here we study the stabilization of saddle point of coupled systems by taking all values of damping parameter *b*. In this context, detection and controlling both saddle and nonsaddle types of unstable steady states in high-dimensional nonlinear dynamical systems based on fast-slow manifold separation and Markov chain theory is articulated in ref. 46.

**Figure 1:**
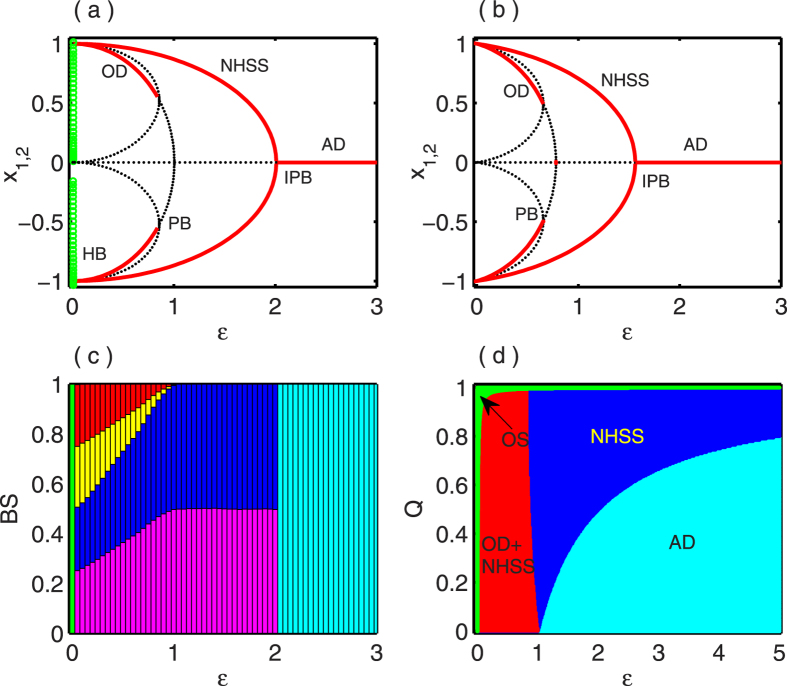
Two coupled Duffing-Holmes oscillators: bifurcation diagram with respect to coupling strength *ε* for (**a**) b = −0.01, (**b**) b = 0.5 where extreme values of *x*_1_ and *x*_2_ are plotted with coupling strength for *Q* = 0.5. Red lines correspond for stable steady states, black dotted points are unstable steady states and green circle for oscillation state. PB: pitchfork bifurcation, OD: oscillation death, AD: amplitude death, IPB: inverse pitchfork bifurcation. (**c**) Variation of BS for different values of coupling strength *ε*. The color green stands for BS of oscillatory state, red and yellow for BS of stable IHSS states *E*_3,4_, blue and magenta for BS of stable NHSS states *E*_1,2_ and color cyan correspond to BS of the HSS state *E*_0_. Other parameters: *b* = −0.01, *Q* = 0.5. (**d**) Two parameter bifurcation diagram in the 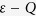 plane where green, red, blue and cyan regions correspond to oscillatory state, coexistence of stable IHSS (OD) and NHSS, stable NHSS state and AD state respectively for b = −0.01

We consider the coupled network through mean-field in the following form:


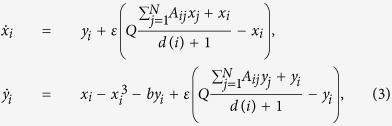


for *i* = 1, …, *N*. Here *ε* is the mean-field coupling strength, *d*(*i*) is the degree of the *i*-th node and 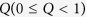 is the mean-field density parameter. This mean-field density parameter *Q* gives an additional free parameter that control the mean-field dynamics while *Q* → 0 represents self-feedback case and *Q* → 1 indicates the maximum mean-field density. The elements of the connectivity matrix *A*_*ij*_ = 1 if *i*-th and *j*-th nodes are connected and zero otherwise. At first we consider a minimal network of two (*N* = 2) coupled Duffing-Holmes oscillators with mean field coupling and identify the parameter region for stabilized saddle point at origin. The coupled DH oscillator has a trivial steady state *E*_0_ = (0,0,0,0) which is the HSS solution of the system and the other four coupling dependent steady states: non-trivial homogeneous steady state (NHSS) 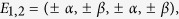 and inhomogeneous steady state (IHSS) 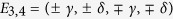 where 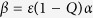, 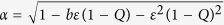, *δ* = *εγ*, 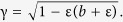 The characteristic equation corresponding to the fixed point *E*_0_ is 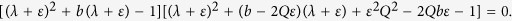 Using Routh Hurwitz(RH) criterion the saddle point *E*_0_ is stable if 
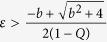
 and that stabilization of saddle point occurred through inverse pitchfork bifurcation. By performing the stability analysis we analytically obtain the inverse pitchfork bifurcation (IPB) point at the coupling strength 
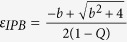
. From linear stability analysis we also analytically derive the Hopf bifurcation (HB) point at 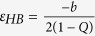 where up to this critical value of the coupling strength, coupled systems exhibit oscillatory states (Fig. 1(a)). Further increment of the coupling strength leads to co-existence of IHSS and NHSS up to a certain threshold of interaction strength 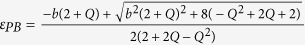 and after *ε*_*PB*_, IHSS are completely eliminated and only NHSS sustained up to *ε*_*IPB*_. So, using linear stability analysis and combining the above results, structurally different dynamical states occur: AD exist for 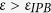, IHSS and NHSS (OD) coexist for 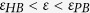 and only NHSS exist for 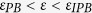.

For numerical simulation, we choose the damping coefficient *b* = −0.01 for which an isolated oscillator exhibits oscillatory dynamics. At lower value of coupling strength *ε*, four coupling dependent fixed points (i.e. NHSS and IHSS) that arise through Hopf bifurcation at *ε* = *ε*_*HB*_, are stable. But as *ε* increases, two of these stable steady states *E*_3,4_ become unstable at 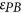 and *E*_12_ remains stable for the value of *ε* upto *ε*_*IPB*_. At *ε*_*IPB*_, saddle point 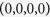 turns stable through IPB and remains stable for *ε* > *ε*_*IPB*_. The corresponding bifurcation diagram (using XPPAUT^47^) is shown in Fig. 1(a). Figure 1(b) shows the bifurcation diagram with respect to coupling strength *ε* when *b* = 0.5, for which an isolated oscillator approaches either to the steady state (1, 0) or to (−1, 0) where for negative values of *b* different coupling dependent stable steady states appear through oscillatory states as shown in Fig. 1(a). Here again, due to the introduction of coupling *ε*, above mentioned four fixed points *E*_1,2_ and *E*_3,4_ become stable but *E*_3,4_ remain stable only upto 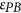. Further increment in the value of *ε* makes the saddle point (0, 0, 0, 0) stable through an inverse pitchfork bifurcation at 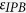. Figure 1(c) shows how the BS of the steady states *E*_1,2_ and *E*_3,4_ change for different values of 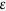. As can be seen, initially after the occurrence of Hopf bifurcation at 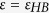, all the fixed points (*E*_1,2,3,4_) are equally probable although the probabilistic dominance of *E*_3,4_ are shrinking gradually whereas *E*_1,2_ acquire more and more space in the basin volume. Such changes on BS measure of *E*_3,4_ gives a hint of the annihilation of *E*_3,4_ which finally occurs at 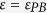 where *E*_1_ and *E*_2_ share the basin with equal probability. But at 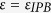, BS of these steady states abruptly decrease to zero without any presage and further increase of *ε*, the basin volume is fully covered by this HSS shown by cyan color with maximum BS i.e., 1. From this figure, we conclude that the BS for multi stable states (i.e. OD and NHSS) change with the variation of mean-field coupling strength *ε* while the BS for monostable state i.e. AD state remains unchanged with the variation of *ε*. Therefore, the trend in the changes of the percentage of the basin volume gives us a clear idea how the different steady states are evolving in a coupled system and which states will dominate the system and which will disappear early. We also obtain similar results on stabilization of saddle point in two coupled DH oscillators when they are coupled through cross mean-field type configuration (see Supplementary Information section I). Figure 1(d) represents the parameter region in 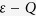 plane where green, red, blue and cyan regions respectively resemble the oscillatory state, coexistence of stable IHSS (OD) and NHSS, stable NHSS state and AD state for *b* = −0.01. For increasing values of *ε* firstly the coupling dependent fixed points get stabilized for almost all the values of *Q* below the Hopf bifurcation curve 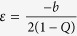. Then the saddle point *E*_0_ becomes stable resulting in AD below the inverse pitchfork bifurcation curve 
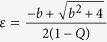
.

We know that the presence of noise is common in real systems. To study the impact of noise in the steady states we use additive Gaussian noise in the system and find that systems still evolve around the steady states with small fluctuations which further implies that BS of each fluctuated steady states does not alter or vanish in the presence of noise. For detailed numerical observations see the Supplementary Information section II.

### Global network of Duffing-Holmes oscillators

Next we check the stabilization of saddle point in a network of equation (3) for higher values of *N* > 2 where *A_ij_* = 1, and *A_ii_* = 0, for all *i*,*j* = 1,2, ...,*N*. At first we start with a complete graph of size N. Fixed points of *N* coupled oscillators are 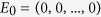, 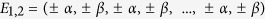, and 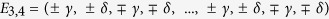 (for even number oscillators) where 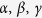 and 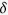 are same as above. The fixed points *E*_1,2_ are same for any choice of *N* whereas *E*_3,4_ are same only for even number of *N*. Characteristic equation at *E*_0_ is


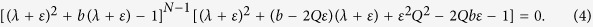


The distinct eigenvalues and critical bifurcation points of globally connected network (3) are same as for two coupled oscillators.

**Figure 2:**
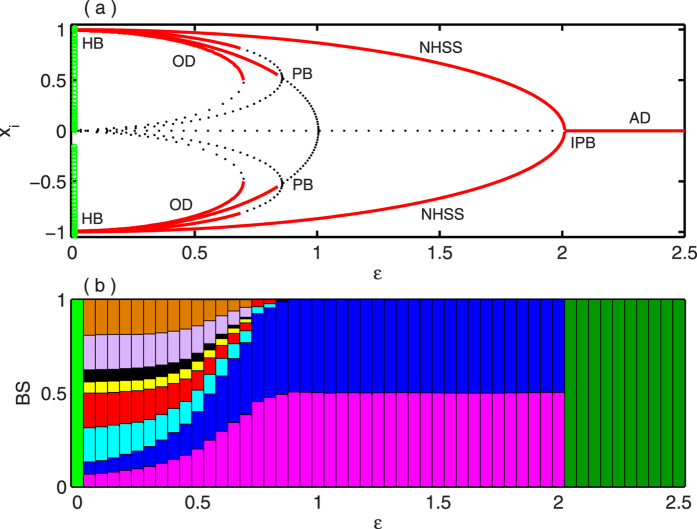
Four coupled Duffing-Holmes oscillators: (**a**) bifurcation diagram with respect to coupling strength *ε* for *b* = −0.01 and *Q* = 0.5, where extreme values of 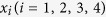 are plotted with coupling strength. Red lines correspond to stable steady states, black dotted points are unstable steady states and green circle for oscillation state. (**b**) Variation of BS for different values of coupling strength *ε*. The color green is for BS of oscillatory state, blue and magenta for BS of stable NHSS states, deep green for BS of stable saddle point *E*_0_ (AD state) and other colors correspond to BS of different stable IHSS states.

First, we consider *N* = 4 i.e. four globally coupled DH oscillators via mean-field coupling and the results are shown in Fig. 2. Analytically it is not easy to calculate all the coupling dependent fixed points (i.e. IHSS and NHSS), using bifurcation diagram (performed in XPPAUT^47^) we identify all the fixed points and by BS measurement we measure the amount of their stability for different values of coupling strength. In Fig. 2(a), bifurcation diagram for the variables 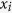 with respect to the coupling strength *ε* is plotted. For small values of *ε*, through Hopf bifurcation at 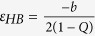, eight coupling dependent fixed points are stable. But as *ε* is increased, firstly six of these stable fixed points lose their stability, among them two lose their stability through PB at 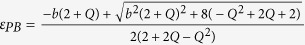 and remaining four lose their stability at earlier of *∈*_*PB*_ and only two retain their stability. Even more increment in *ε* makes the two fixed points unstable and the saddle point (i.e., the origin) becomes stable through IPB. The process of stabilization and destabilization of all the coupling dependent fixed points are clarified in terms of their BS which validates the whole mechanism in global scale. Figure 2(b) shows the variation of BS for different steady states by varying the mean-field coupling strength *ε*. As mentioned earlier, the blue and magenta color in Fig. 2(b) belong to class NHSS and they acquire more and more space in the basin if we increase the coupling strength continuously. On the other hand, the other cluster belonging to IHSS (six states in three symmetric groups) losing their stability and finally all of them vanish at 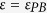 point. Further changes in *ε* makes those two fixed points (NHSS) equally probable in the basin i.e. each of them acquires half of the whole basin and they become unstable at the point 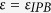. Then the saddle point (i.e. the origin) becomes stable for all points in the basin of attraction i.e. the basin volume is fully covered by this HSS.

**Figure 3:**
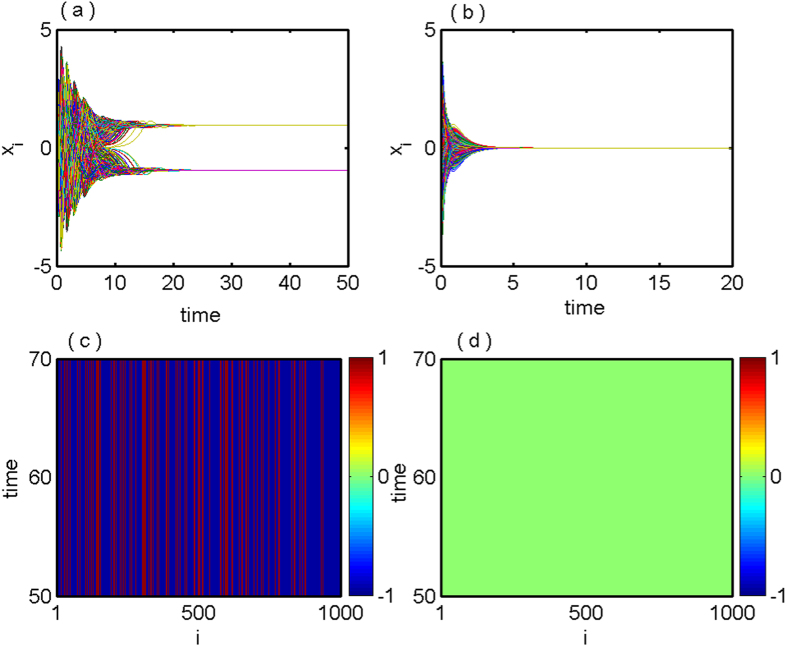
Globally coupled Duffing-Holmes oscillators for *N* = 1000: (**a**) time series of *x*_*i*_, (*i* = 1, 2, …, 1000) show the IHSS state for *ε* = 0.3. (**b**) Time series of stabilized saddle point *E*_0_ for *ε* = 2.2. (**c**) and (**d**) corresponding space-time plot of (**a**) and (**b**) respectively showing stable IHSS and HSS states. Other parameters are: *Q* = 0.5, *b* = −0.01.

Next we will verify numerically whether the stabilization of saddle and all the coupling dependent steady states using the proposed coupling scheme is working in a large network. For our case, we choose *N* = 1000 globally coupled DH oscillators via mean-field and the analyzed results are illustrated in Fig. 3. Figure 3(a) shows time series of *x*-components of all the 1000 oscillators with *ε* = 0.3 that depicts the stabilization of the IHSS resulting in OD. For larger value of *ε (ε* = 2.2), all the oscillators populate to a single steady state, that is, the saddle point (the origin) gets stabilized, time series are shown in Fig. 3(b). The corresponding space-time plots are shown in Fig. 3(c) and (d) respectively. The parameter space in 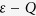 plane for global network is same as in Fig. 1(d) for two coupled DH oscillators, as the distinct eigenvalues of the characteristic equation (4) are identical with two coupled case but with different multiplicity.

### Random network of Duffing-Holmes oscillators

**Figure 4:**
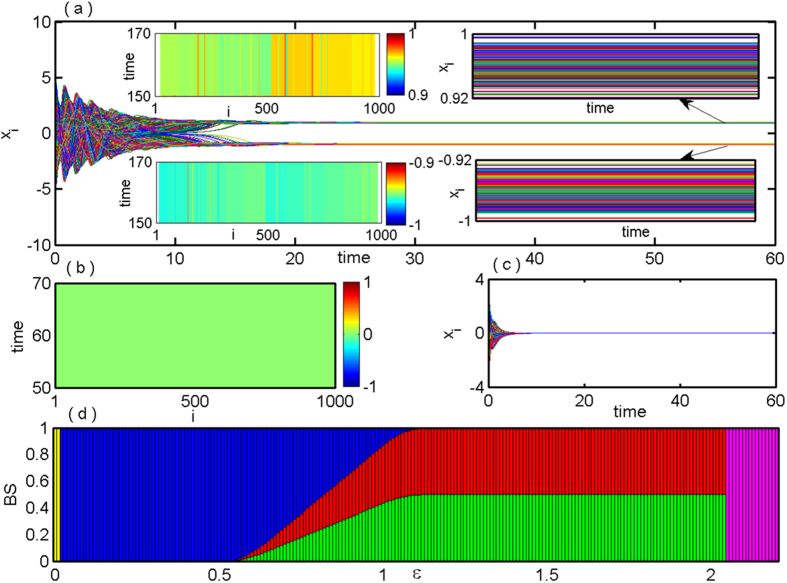
Randomly coupled Duffing-Holmes oscillators (*N* = 1000): (**a**) time series of *x*_*i*_, (*i* = 1, 2, …, 1000) show the MCOD state for *ε* = 0.3. Right and left inset figures in (**a**) show the time series of coupling dependent different steady stables and corresponding spatio-temporal plots respectively. (**b**) Space-time plot and (**c**) corresponding time series of stabilized saddle point *E*_0_ for *ε* = 3.0. (**d**) BS of MCOD, NHSS and AD states against the coupling strength *ε*. The oscillatory state, MCOD, NHSS and AD states are represented by yellow, blue, red/green and magenta colors respectively. Other parameters are: *Q* = 0.5, *b* = −0.01.

In this section we investigate the stabilization of saddle point in Erdös-Rényi random networks of DH oscillators and the results are shown in Fig. 4 where the probability of existence of an edge between any two vertices of the random network is taken as *p* = 0.01. Figure 4(a) shows the time series of the 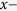
components of all the *N* = 1000 oscillators characterizing MCOD state for *ε* = 0.3. The inset figures (right panel) show the magnified time-series plots for better visibility of the MCOD state. The space-time plots corresponding to these time-series are given in the insets (left panel) of Fig. 4(a). Figure 4(b) and (c) show the space-time plot and the corresponding time series represent stabilization of the saddle point (the origin) resembling AD state for *ε* = 3.0. Figure 4(d) depicts the variation of the BS of the MCOD and NHSS states for the random network. Due to failure of calculation of all the MCOD states analytically in a random network, we consider all the states as MCOD state and represented by blue color in Fig. 4(d). After the Hopf bifurcation, MCOD state dominates over the NHSS state where relative acceptance of MCOD in BS measure is almost unity and the probability of occurrence of NHSS is almost nil. With increasing the values of the coupling strength *ε*, the probability of getting NHSS state increases and MCOD state decreases. Then the BS of NHSS starts increasing gradually and vanishing of BS of the MCOD state is observed for 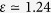. NHSS remains stable further upto 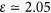 from where the saddle point becomes stable through IPB with BS unity.

### Lorenz oscillators

For quantifying the different stable steady states using BS measure, we extend our investigation on coupled paradigmatic chaotic Lorenz oscillator^48^. We consider *N* Lorenz oscillators interacting through mean-field diffusive coupling. The mathematical equations of the coupled systems are described as:


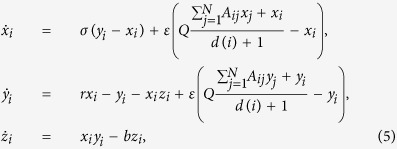


for *i* = 1, 2, 3, …, *N*. In absence of coupling term, each oscillators oscillate chaotically for 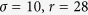 and 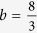 and the individual systems have a saddle fixed point at origin and two unstable fixed point at 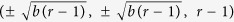. Here *ε* and *Q* are the coupling strength and mean-field density parameter respectively.

For *N* = 2, the fixed points are 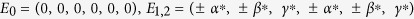, where 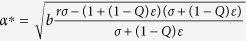, 
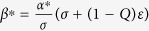
 and 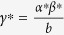. The characteristic equation at *E*_0_ is


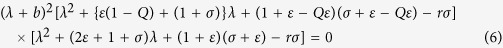


The trivial fixed point *E*_0_ is stable through inverse pitchfork bifurcation at 
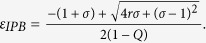


**Figure 5:**
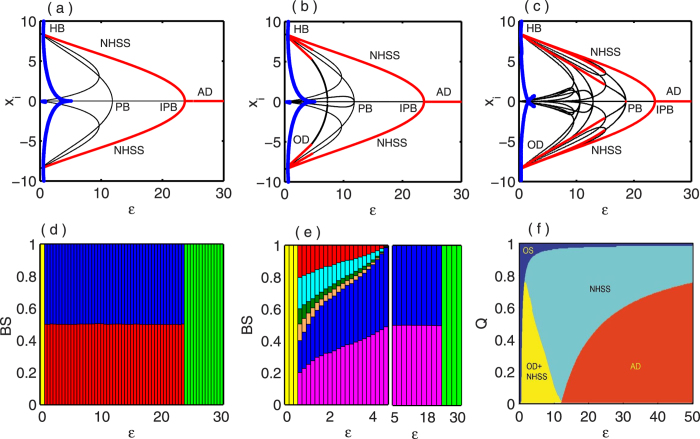
Coupled Lorenz oscillators: Bifurcation diagrams by changing the coupling strength *ε* for (**a**) *N* = 2 and (**b**) *N* = 4 globally coupled oscillators. (**c**) Bifurcation diagram for randomly coupled *N* = 4 oscillators, (d) For *N* = 2 and (**e**) *N* = 4 globally coupled oscillators, the variation of BS with respect to coupling strength *ε* Other parameter *Q* = 0.5. (f) Parameter region in 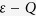 plane for *N* = 4 globally coupled network.

For fixed values of the above system parameters, from eigenvalue analysis the NHSS points *E*_1,2_ becomes stable for 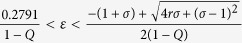. The results are shown in Fig. 5. Figures 5(a) and (b) show bifurcation diagrams with respect to *ε* for *N* = 2 and *N* = 4 respectively with *Q* = 0.5 fixed. As in Fig. 5(a), due to the presence of coupling, two stable fixed points *E*_1,2_ develop together with six unstable fixed points through Hopf bifurcation at 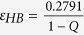 and *E*_1,2_ remain stable for *ε* upto 
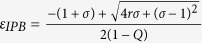
. The saddle point *E*_0_ becomes stable through an inverse pitchfork bifurcation at 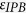, and persists for any higher values of *ε* as well. For *N* = 4, Fig. 5(b) shows that immediately after the occurrence of Hopf bifurcation at 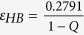, six coupling dependent stable fixed points (comprising of both IHSS and NHSS states) emerge together with six unstable fixed points. But among them, the fixed points except the NHSS *E*_1,2_ lose their stability soon and only *E*_1,2_ remain stable for higher values of *ε*. Similarly as before, through IPB at 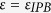, *E*_1_ and *E*_2_ collides and *E*_0_ turns stable. Figure 5(c) shows the bifurcation diagram against *ε* for a network of *N* = 4 randomly connected nodes where the appearance of six coupling dependent stable fixed points along with many other unstable fixed points can be seen. Similarly as in the previous cases, here also *E*_1,2_ retain their stability for higher values of *ε* than the others and lose it stability at 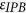 and further higher coupling strength promotes the entire systems to the AD state. Figures 5(d) and (e) measure all the stable steady states that appear for *N* = 2 and *N* = 4 respectively in terms of their BS. Figure 5(d) shows that the BS of both *E*_1_ and *E*_2_ are non-zero and more or less the same for all values of *ε* upto 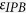. As *ε* increases further, BS of *E*_1_ and *E*_2_ turns into zero and BS of *E*_0_ becomes unity. On the other hand, soon after the Hopf bifurcation all the six coupling dependent stable fixed points get non-zero BS but *E*_1_ and *E*_2_ have larger BS than the others, as in Fig. 5(e) (left part). Increasing *ε*, BS of the other fixed points become zero and *E*_1_ and *E*_2_ shares almost the same BS value upto 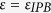. After that BS of both *E*_1,2_ becomes zero and that of *E*_0_ appears to be 1 (right part in Fig. 5(e)).

Finally, Fig. 5(f) depicts the parameter region in 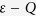 plane for globally coupled *N* = 4 oscillators. Here blue, yellow, cyan and red regions signify oscillatory state, co-existence of OD and NHSS states, stable NHSS state and AD state (i.e., the stabilization of saddle *E*_0_) respectively. The oscillatory state (blue region) and coexistence of OD and NHSS (yellow region) or stable NHSS (cyan region) are separated by the Hopf bifurcation curve 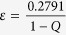. From this curve it is clear that the oscillatory state persists for higher values of coupling strength *ε*. The stability of OD or NHSS loses when the value of *ε* passes through the inverse pitchfork bifurcation curve 
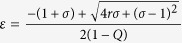
.

### Networks of Lorenz oscillators

Next we will explore the proposed coupling scheme is applicable for large number of chaotic oscillators. To quantify the stability of different steady states using BS measure in global and random network. The characteristic equation at *E*_0_ of network (5) is


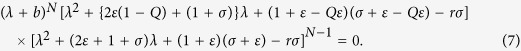


**Figure 6:**
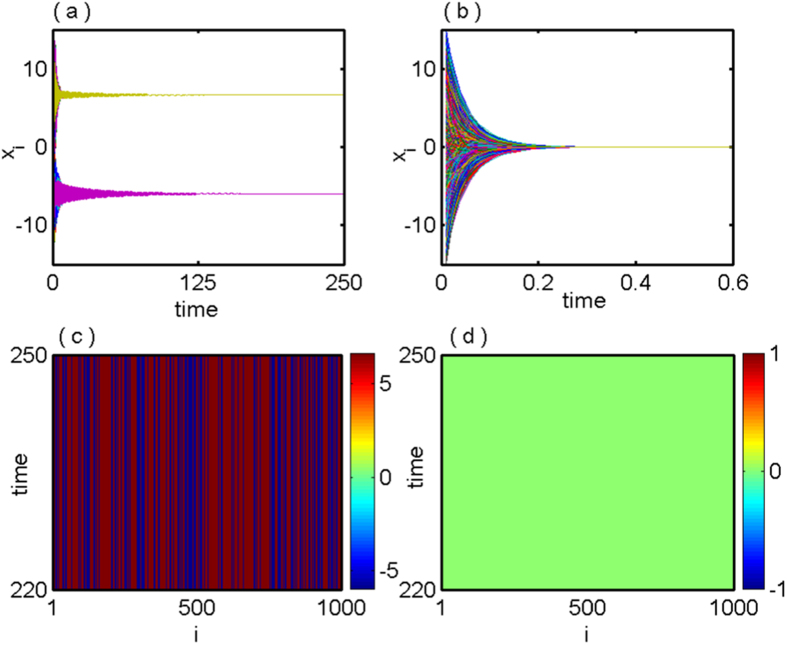
Global network of Lorenz oscillators: (**a**) and (**b**) show the time series of IHSS and HSS states for coupling strength *ε* = 5.5 and *ε* = 30 and (**c**), (**d**) represents the corresponding space-time plot of (**a**) and (**b**) respectively. Here *N* = 1000 and *Q* = 0.5.

Taking a network of *N* = 1000 globally coupled Lorenz oscillators with *Q* = 0.5, the numerical results are shown in Fig. 6. Figure 6(a) shows time evolution of the 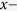
components of all the 1000 oscillators with *ε* = 5.5 that represents the stabilization of IHSS resembling OD. Whereas for *ε* = 30, the saddle point (origin) appears to be stable, time series shown in Fig. 6(b). Figure 6(c) and (d) depict the corresponding space-time plots respectively.

**Figure 7:**
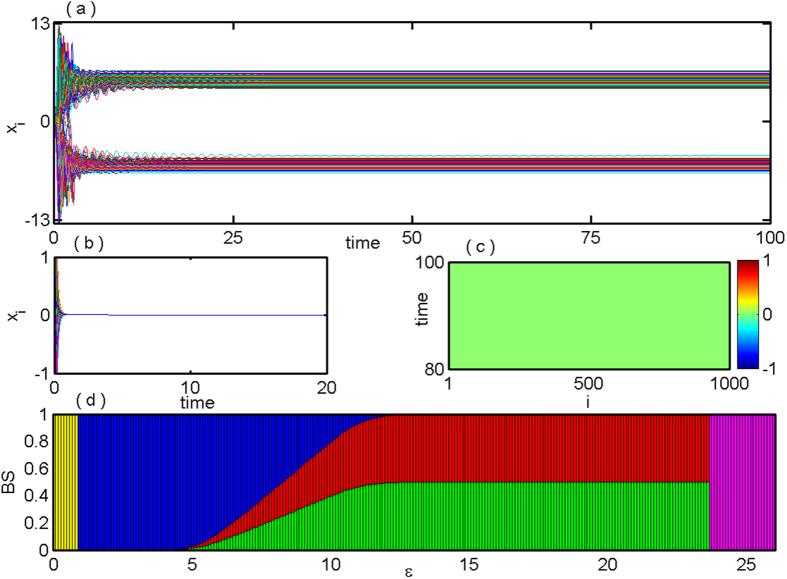
Random network of Lorenz oscillators: (**a**) Time series of *x*_*i*_, (*i* = 1, 2, …, 1000) shows MCOD state for *ε* = 6.0 (**b**) Time series of *x*_*i*_, (*i* = 1, 2, …, 1000) show the stabilized saddle state for *ε* = 30.0 and (**c**) corresponding space-time plot. (**d**) Variation of BS with respect to the coupling strength *ε* where yellow color represents oscillatory behaviors, blue for MCOD, red and green for corresponding NHSS states and magenta for AD state. Other parameter fixed at *Q* = 0.5 and *N* = 1000.

Results regarding MCOD state and saddle stabilization in Erdös-Rényi random networks of coupled Lorenz systems are given in Fig. 7. Time series of the 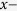
components of all the *N* = 1000 oscillators revealing MCOD state for *ε* = 6.0 and *Q* = 0.5 are shown in Fig. 7(a). In Fig. 7(b) stable AD state ensuing after a concise transient window and Fig. 7(c) shows the corresponding space-time plots representing stabilization of the saddle point (the origin) reflecting AD for 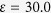 and 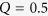. The dependence of the BS of the MCOD and NHSS states on coupling strength *ε* for the random network of Lorenz systems is characterized in Fig. 7(d). Here, after the Hopf bifurcation at 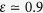, the MCOD state retains BS almost 1 and the BS of NHSS is very small upto 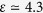. In fact, for 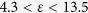 the states MCOD and NHSS co-exist but then BS of NHSS develops with tantamount and that of the MCOD state vanishes at 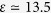 and NHSS remains stable further upto 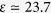 from where the saddle point becomes stable abruptly without any pre-warning and carries BS unity further.

## Discussion

In this work we have studied basin stability (BS) measure to quantify the stability of different stable steady states of coupled dynamical systems interacting through mean-field coupling. BS is an universal concept to quantify the stability of governing dynamical systems under a non-uniform distribution of perturbations. Using mean-field coupling configuration, we have obtained a homogeneous stable steady state (i.e. AD state) which is inherently saddle equilibrium point of the individual oscillator and also showed that the transition from inhomogeneous steady states (resembling OD) to homogeneous steady state (i.e. AD state) via stabilization of NHSS state. We identify the underlying mechanism to stabilize the saddle fixed points in a network of coupled oscillatory systems. The transition routes between different states of coupled systems are discussed through rigorous bifurcation analysis and confirmed with the obtained analytical results. We also map the different steady states in the wide parameter space by varying the mean-field coupling strength *ε* and mean-field density parameter *Q*. All the steady states are quantified by the value of BS. In contrast to this, we found that the BS of OD states gradually decreases as coupling strength increases. After annihilation of the BS of multi-stable OD states, the BS of NHSS states become prevalent with almost equal ratio. But further increasing of coupling strength NHSS states become unstable without any presage and immediately AD state is stabilized. In the context of oscillations suppression studies, all the previous works have been done by considering the specific initial conditions in the phase space and no one examined the whole basin volume therefore ignoring the multistability nature of the steady states. As multistable character is ubiquitous in natural systems so we clearly elucidate a global stability measure by means of basin stability. To validate the BS measure, we have considered a large number of initial states following^16^. All these phenomena and measures are performed using smaller size of networks (for N = 2 and N = 4) as well as network of bigger size (N = 1000). We test our proposition and statistical measure not only in complete graph but also in random network. For both cases, our analytical and numerical simulations give proper insight to track the multistablity features present in the systems. The models considered here cover the characteristics of limit cycle (Duffing-Holmes oscillator) or chaotic attractor (Lorenz system) having hyperbolic fixed points. There are many real systems such as laser^49^ and geomagnetic^50^ which are modeled like Lorenz systems or mimic of Lorenz systems after some transformations and the results of our approach can be easily implemented.

Our considered mean-field coupling is one of the most natural coupling scheme which is previously extensively applied to different branches of science and engineering. This strongly means that our approach is not limited to a particular situation or for some particular systems, rather this mechanism is applicable in wide range of systems throughout all these disciplines. Also multistable feature is omnipresent in nature and widespread phenomenon in dynamical systems that appears in diverse fields ranging from physics, chemistry, biology to social systems^51^. There are numerous systems in which multistability originates that include the human brain, semiconductor materials, chemical reactions, metabolic system, arrays of coupled lasers, hydrodynamical systems, various ecological systems, artificial and living neural systems etc. We believe that this study will broaden our understanding of stabilization of saddle points in multistable dynamical networks where units are connected via mean-field. Further we have shown that the critical mean-field coupling strength is independent of the size of the network but only depends on the largest real part of the eigenvalue of individual oscillator (refers to Linear Stability Theorem in Method Section).

## Methods

### Basin Stability Measure

Let *I* be the set of initial values for a given coupled system of *N* oscillators which is a bounded subset of 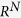. Suppose 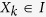 is an asymptotically stable equilibrium point of the given system. Now let 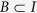 be the basin of attraction of the stable state *X*_*k*_ (i.e, the solution of the system starting from any 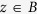 asymptotically converges to *X*_*k*_ as *t* → ∞).

We numerically integrate the given system for *V* points which are drawn uniformly at random (sufficiently large) from *I*. Let *V*_*k*_ be the count of the initial conditions that finally arrives at the stable steady state *X*_*k*_. Then the BS for the fixed point *X*_*k*_ is estimated as 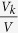.

### Numerical Simulation

For numerical integration, we used fifth-order Runge-Kutta-Fehlberg algorithm with fixed step size Δ*t* = 0.01. For simulations of BS measure we choose sufficiently large number (for regular networks 20000 and for irregular networks, 5000) of initial conditions and all random initial conditions are chosen from [−5, 5] × [−5, 5] for coupled Duffing-Holmes oscillators and [−20, 20] × [−30, 30] × [0, 50] for coupled Lorenz oscillators.

### Linear Stability Theorem

If 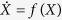 be 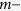
dimensional dynamical system which exhibits a saddle equilibrium point *O*, the saddle equilibrium point can be stabilized in globally mean-field coupled of N identical systems and the critical coupling strength is 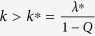, where λ* is the maximum real part of eigenvalues of the isolated system at the equilibrium point *O* and 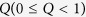 is the mean-field density parameter.

**Proof:** Consider N identical systems interacting through global mean-field diffusive coupling as follows:


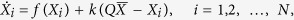


where *f*(*X*_*i*_) be the evolution equation of the *i*^*th*^ system, *X*_*i*_ denotes 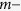
dimensional state vector, *k* be the mean-field coupling strength, *Q* is the mean-field density parameter and 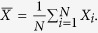


The isolate system 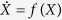 possess a saddle equilibrium point *O*. So the Jacobian matrix 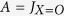 of this system has at least two real eigenvalues with opposite sign. Let λ* be the maximum real part of eigenvalues 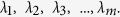


The Jacobian matrix of the above coupled systems at the trivial equilibrium point 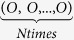 is


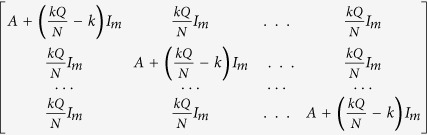


The corresponding *characteristic equation* is


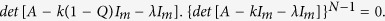


The eigenvalues are 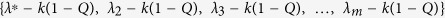 and 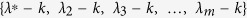 (*N* − 1) times. The saddle point *O* is stable if all the real parts of the eigenvalues are negative negative. For this it is sufficient to make 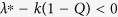. From this we have the critical coupling strength is 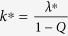.

## Additional Information

**How to cite this article**: Rakshit, S. *et al*. Basin stability measure of different steady states in coupled oscillators. *Sci. Rep.* **7**, 45909; doi: 10.1038/srep45909 (2017).

**Publisher's note:** Springer Nature remains neutral with regard to jurisdictional claims in published maps and institutional affiliations.

## Supplementary Material

Supplementary Information
