# Supplementary material for: Basin stability measure of different steady states in coupled oscillators
Source: Sci Rep. 2017 Apr 5;7:45909. doi: 10.1038/srep45909 (PMC5381114; doi:10.1038/srep45909)
Supplement: Supplementary Information [file srep45909-s1.pdf]

Basin stability measure of different steady states in coupled oscillators

Supplementary Information

Sarbendu Rakshit,[1] Bidesh K. Bera,[1] Soumen Majhi,[1] Chittaranjan Hens,[2] and Dibakar Ghosh[1,*]

[1] *Physics and Applied Mathematics Unit, Indian Statistical Institute, Kolkata-700108, India*

[2] *Department of Mathematics, Bar-Ilan University, Ramat Gan 52900, Israel*

*Electronic address: diba.ghosh@gmail.com

I. CROSS MEAN-FIELD INTERACTION

We briefly discuss the different steady states in two coupled Duffing-Holmes oscillators interacting through cross mean-field coupling. We calculate the equilibrium points and derive the critical coupling strength using linear stability analysis. Numerical simulations confirm the analytical results and the probability of initial conditions for approaching different steady states using basin stability (BS) measure are calculated.

We consider two coupled Duffing-Holmes oscillators interacting through the cross mean-field coupling and the mathematical form as follows:

$$\begin{aligned}
\dot{x}_1 &= y_1 + \epsilon(Q\tfrac{y_1+y_2}{2} - x_1),\\
\dot{y}_1 &= x_1 - x_1^3 - by_1 + \epsilon(Q\tfrac{x_1+x_2}{2} - y_1),\\
\dot{x}_2 &= y_2 + \epsilon(Q\tfrac{y_1+y_2}{2} - x_2),\\
\dot{y}_2 &= x_2 - x_2^3 - by_2 + \epsilon(Q\tfrac{x_1+x_2}{2} - y_2),
\end{aligned} \tag{1}$$

where ϵ is the cross mean-field coupling strength and $Q(0 \leq Q < 1)$ is the mean-field density parameter, as stated in the main text. The above coupled equation has the following fixed points:
(i) trivial steady state $E_0 = (0,0,0,0)$ which is the homogeneous steady state (HSS) solution of the system,
(ii) two coupling dependent steady states $E_{1,2} = (\alpha, \beta, \alpha, \beta)$ where $\alpha = \frac{1+\epsilon Q}{\epsilon}\beta$ and $\beta = \pm\sqrt{\frac{\epsilon^2}{1+\epsilon Q} - \frac{(b+\epsilon)\epsilon^3}{(1+\epsilon Q)^3}}$. This steady state corresponds to the non-trivial homogeneous steady state (NHSS). The other coupling dependent steady state is
(iii) $E_{3,4} = (\gamma, \delta, -\gamma, -\delta)$ where $\gamma = \pm\sqrt{1-(b+\epsilon)\epsilon}$ and $\delta = \pm\epsilon\sqrt{1-(b+\epsilon)\epsilon}$. This state corresponds to the inhomogeneous steady state (IHSS).

The eigenvalues corresponding to the steady state E_0 of the coupled system are

$$\lambda_{1,2} = \frac{-(b+2\epsilon) \pm \sqrt{b^2+4}}{2}$$

and

$$\lambda_{3,4} = \frac{-(b+2\epsilon) \pm \sqrt{b^2+4\epsilon^2Q^2+8\epsilon Q+4}}{2}$$

From the eigenvalue analysis we derive the hopf bifurcation point (HB) at the coupling strength $\epsilon_{HB} = -\frac{b}{2}$, the inverse pitchfork bifurcation point (IPB) at the coupling strength $\epsilon_{IPB} = \frac{-(b-2Q)+\sqrt{(b-2Q)^2+4(1-Q^2)}}{2(1-Q^2)}$. The origin is stable if $\epsilon > \frac{-(b-2Q)+\sqrt{(b-2Q)^2+4(1-Q^2)}}{2(1-Q^2)}$. The equilibrium points $E_{1,2,3,4}$ emerge at $\epsilon = \epsilon_{HB}$ through HB. The steady state E_{NHSS} is stable if

$-\frac{b}{2} < \epsilon < \frac{-(b-2Q)+\sqrt{(b-2Q)^2+4(1-Q^2)}}{2(1-Q^2)}$. Using eigenvalue analysis, the symmetry breaking coupling dependent steady state $E_{3,4}$ is stable if $b + 2\epsilon > 0, \epsilon(b + \epsilon) - k_2(1 + \epsilon Q) > 0$ where $k_2 = \epsilon Q - 2 + 3\epsilon(b + \epsilon)$. From eigenvalue analysis, we derive the Hopf bifurcation curve as $b + 2\epsilon = 0$ and inverse pitchfork bifurcation curve as $\epsilon^2(1 - Q^2) + \epsilon(b - 2Q) - 1 = 0$.

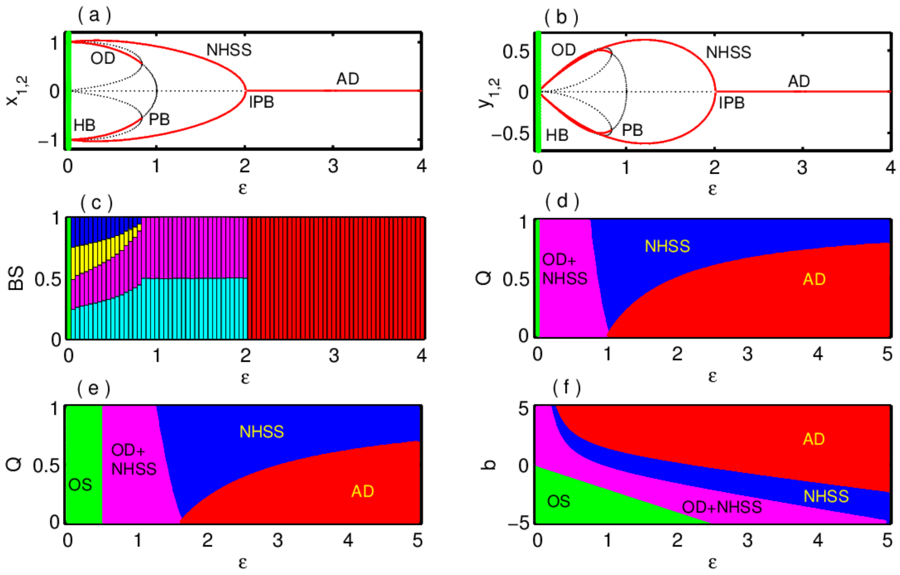

FIG. 1: Two Duffing-Holmes oscillators coupled through cross mean-field coupling: bifurcation diagrams by varying the cross mean-field coupling strength ϵ where extrema of (a) $x_{1,2}$ and (b) $y_{1,2}$ are plotted for $b = -0.01$ and $Q = 0.5$. (c) Variation of BS for different values of ϵ where regions of color green represents oscillatory state, cyan and magenta for the steady states $E_{1,2}$, blue and yellow for the steady states $E_{3,4}$ and red for amplitude death state. Two parameter bifurcation diagrams in the $\epsilon - Q$ plane for (d) $b = -0.01$ and (e) $b = -1.0$. (f) Two parameter bifurcation diagram in the $\epsilon - b$ plane for $Q = 0.5$. The region of green, magenta, blue and red corresponding for the oscillatory, coexistence of OD and NHSS, solely NHSS and AD states respectively.

Figures 1(a) and (b) show the bifurcation diagrams with respect to coupling strength ϵ corresponding to the $x-$ and $y-$ components respectively with $b = -0.01$ and $Q = 0.5$. As reflected in both of these figures, different coupling dependent fixed points, namely $E_{1,2}$ and $E_{3,4}$ resembling NHSS and OD states appear from oscillatory state through Hopf bifurcation at ϵ_{HB}. Further increment in ϵ gives rise to an inverse pitchfork bifurcation at ϵ_{IPB} through which the saddle point (the origin) gets stabilized that signifies the AD state. The variation in the BS of the steady states $E_{1,2}$ and $E_{3,4}$ with coupling strength ϵ are depicted in Fig. 1(c). For very small value of ϵ, after the

occurrence of Hopf bifurcation at ϵ_{HB}, initially all the fixed points $E_{1,2}$ and $E_{3,4}$ share almost the same BS but as ϵ increases BS of $E_{3,4}$ starts decreasing and becomes zero at ϵ_{PB}. From then the BS of $E_{1,2}$ remains same and further abruptly turns into zero at ϵ_{IPB}. Further hike in ϵ leads the BS of saddle point E_0 to be unity. $Q = 0.5$ and $b = -0.01$ are kept fixed in this case.

Figure 1(d) represents the parameter region in $\epsilon - Q$ plane in which green, magenta, blue and red regions respectively correspond to the oscillatory state, coexistence of OD and NHSS, stable NHSS state and AD state for $b = -0.01$. As can be seen, here oscillation exists for all values of Q but for a very narrow range of ϵ only. At the right of the Hopf bifurcation curve $b + 2\epsilon = 0$, firstly coupling dependent fixed points get stabilized and then AD state come out depending on the value of Q. However, we choose the value of b smaller than that used in Fig. 1(d), namely we take $b = -1.0$ and plot the $\epsilon - Q$ parameter plane in Fig. 1(e). As expected, because of the form the HB curve $b + 2\epsilon = 0$, a broader range of ϵ is now indicating oscillation of the coupled system in color green, in this case. The other rengions of coexistence of OD and NHSS, stable NHSS state and AD state are plotted in magenta, blue and red colors respectively as before.

Finally we plot the $\epsilon - b$ parameter plane in Fig. 1(f) while keeping $Q = 0.5$ fixed. Only the negative values of b can produce oscillation as shown in the figure. The process of stabilization of saddle point implying AD state through the stabilization of other coupling dependent fixed points indicating OD and NHSS state for almost all the values of b is visible here.

II. EFFECT OF NOISE ON STEADY STATES

In the main text, we analyze the stability and probabilistic dominance of each steady states. We will analyze here the impact of noise in those steady states. Here we consider two DH oscillator coupled through mean-field coupling with additive common noise at each variable in the following form:

$$\begin{aligned}
\dot{x}_1 &= y_1 + \epsilon(Q\tfrac{x_1+x_2}{2} - x_1) + \xi(t) \\
\dot{y}_1 &= x_1 - x_1^3 - by_1 + \epsilon(Q\tfrac{y_1+y_2}{2} - y_1) + \xi(t) \\
\dot{x}_2 &= y_2 + \epsilon(Q\tfrac{x_1+x_2}{2} - x_2) + \xi(t) \\
\dot{y}_2 &= x_2 - x_2^3 - by_2 + \epsilon(Q\tfrac{y_1+y_2}{2} - y_2) + \xi(t)
\end{aligned} . \tag{2}$$

where ϵ is the mean-field coupling strength, $Q(0 \leq Q < 1)$ is the mean-field intensity, $\xi(t)$ is the Gaussian white noise with the properties $\langle \xi(t) \rangle = 0$ and $\langle \xi(t)\xi(t') \rangle = 2D\delta(t - t')$ where $D > 0$ is the noise intensity, δ is Dirac delta function, and $\langle . \rangle$ denotes averaging over the realizations of

$\xi(t)$.

At first we will prove why stabilization of fixed points (linear stability approach) under noise is nearly impossible. However, numerically we will show that for a broader range of noise interaction the new states oscillate around the old states with a negligible fluctuations therefore the BS remains almost same as before.

Theorem : *Oscillation suppression is impossible for noise induced continuous dynamical system.*

Proof : Consider $\dot{\mathbf{X}} = \mathbf{f}(\mathbf{X}, \mu)$ be $m-$dimensional continuous dynamical system, μ be it's system parameter. Let $\mathbf{X} = \mathbf{X}^*$ be a stable equilibrium point for some value of μ. So the system will converge to the equilibrium point ($\mathbf{X}^*$) for any local perturbation of $\mathbf{X(t)}$ near the equilibrium point i.e. $\dot{\mathbf{X}}(\mathbf{t}) = 0$ as $t \to \infty$. The zero velocity of the system signifies the stabilization of $\mathbf{X}(\mathbf{t})$ at that point.

Now if we introduce additive noise in that system then it follows

$$\dot{\mathbf{X}} = \mathbf{f}(\mathbf{X}, \mu) + B\xi(\mathbf{t}),$$

where B is a diagonal matrix of order m, $\xi(t)$ be the noise function which is time dependent. For an arbitrary initial condition, let's assume the system vector $\mathbf{X}(\mathbf{t})$ arrives at $\mathbf{X}(\mathbf{t_0})$ when $t = t_0$. Then the velocity at the time $t = t_0$ becomes

$$\dot{\mathbf{X}}(\mathbf{t_0}) = \mathbf{f}(\mathbf{X}(\mathbf{t_0}), \mu) + \mathbf{B}\xi(\mathbf{t_0}).$$

Now since $\xi(\mathbf{t})$ fluctuates with time t and it is independent of the evolution function $\mathbf{f}(\mathbf{X}, \mu)$ so $\dot{\mathbf{X}}$ will never be constant for all subsequent time $t > t_0$. Particularly, it will never converges to zero as time increases. The $non - zero$ velocity will force the system to oscillate i.e. $\mathbf{X}(\mathbf{t})$ will never be stabilized as t grows up.

The above mathematical logic emphasizes that oscillation suppression is impossible for noise induced dynamical systems. □

We numerically check the effect of noise in DH oscillators (Eqn. 2). Figure 2 describes the effect of noise on the steady states present in the systems. The system parameters are same (follow the main text). The noise intensity is taken as $D = 0.08$. We check three coupling regimes (ϵ) where the steady states are structurally different. In Fig. 2(a) four states (two NHSS and two IHSS shown in black line) are plotted as a function of time (coupling strength $\epsilon = 1.0$) and we

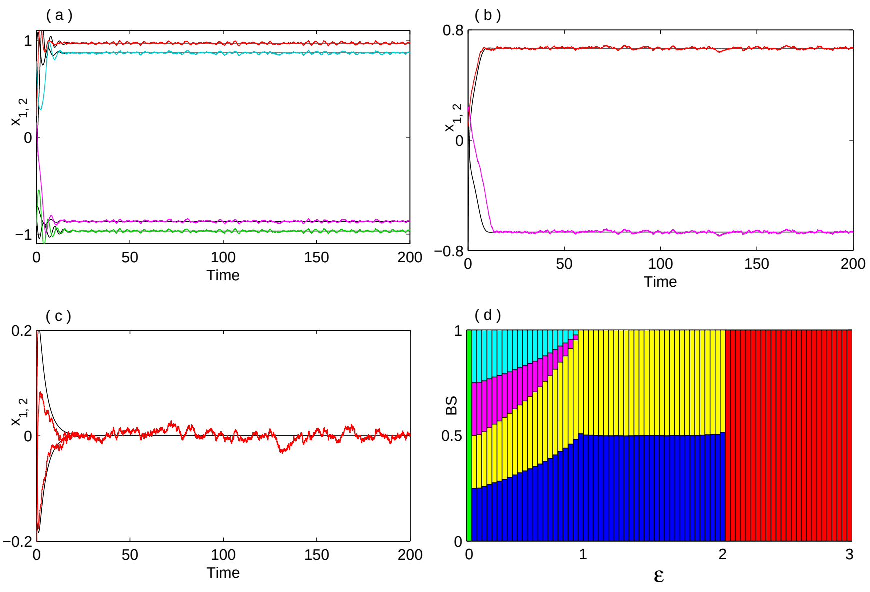

FIG. 2: Two coupled DH oscillator with common noise: Time series of the state variable $x_{1,2}$ for (a) $\epsilon = 0.5$, (b) $\epsilon = 1.5$, (c) $\epsilon = 2.5$ where black straight line shows the time series of steady states of noise-free ($D = 0$) system, other color curves be the time series of the noise induced system for different initial conditions. (d) Variation of BS of these fluctuation state for various value of coupling strength ϵ. The color region green, cyan, magenta, yellow, blue, red corresponds to oscillation state, fluctuations around the steady states E_4, E_3, E_2, E_1 and E_0. Other parameters: $b = -0.01, Q = 0.5$, noise intensity $D = 0.08$.

observe small oscillations (shown in four colors around the steady states (black lines)) due to the presence of noise. Figure 2(b) reveals the nature of NHSS at $\epsilon = 1.5$ where IHSS do not exist. The small oscillations exist around the steady states due to the presence of noise. Further Fig. 2(c) explores the behavior of one steady state: the stabilization of the saddle equilibrium with noisy fluctuation. It seems for all cases the noise effect is statistically negligible as all the time series oscillate around the steady states with negligibly small amplitude. We finally check how the Basin Stability of these fluctuation states appear in the basin volume. Figure 1(d) reveals the BS measure of such fluctuations around the steady states $E_{1,2}, E_{3,4}$ and E_0 as a function of ϵ. The qualitative and quantitative behavior of this BS is exactly same with the BS scenario of the noise-free system. All the bifurcation points (HB, IPB) also appear in the same points.

In the $\epsilon - D$ parameter space, the BS of noise induced fluctuation states around E_4, E_3, E_2, E_1 and E_0 are shown in the color coded Figs. 3(a), (b), (c), (d) and (e) respectively. Figures 3(a),

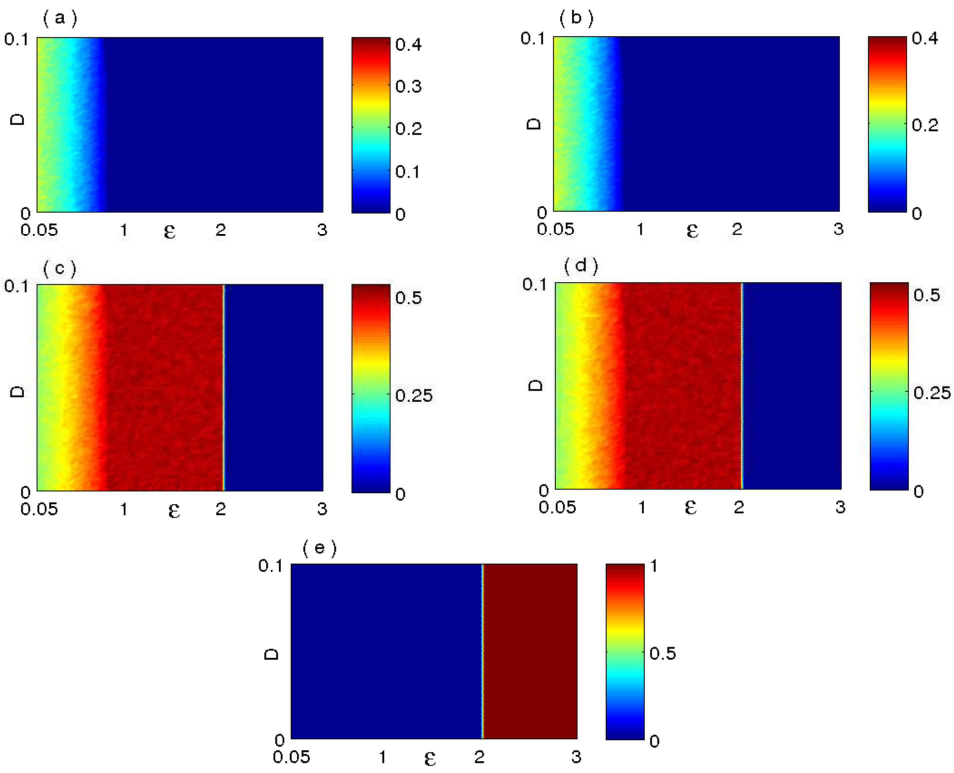

FIG. 3: Coexistence of different fluctuation states around fixed points are quantified by a color bar of basin stability measure in $\epsilon - D$ parameter space of two coupled DH oscillator with common noise; $b = -0.01, Q = 0.5$: where (a), (b), (c), (d) and (e) represent the BS of fluctuated states around E_4, E_3, E_2, E_1 and E_0 respectively.

(b) show that just after the Hopf bifurcation point the BS of noise induced $E_{3,4}$ takes the value almost equal to 0.25 for all $D \in [0, 0.1]$. Actually each of them acquires 25 percent of the whole space because four steady states ($E_{1,2,3,4}$) coexist together. However, the BS of these sates ($E_{3,4}$) gradually decrease for more increasing ϵ, and finally they become unstable at $\epsilon = \epsilon_{PB}$. And we observe that there is no impact of noise intensity on it's BS.

The BS scenario of the fluctuation states around $E_{1,2}$ are shown in Figs. 3(c), (d). After the Hopf bifurcation point they take value approximately 0.25 for any noise intensity ($D \in [0, 0.1]$). Further increase of ϵ the BS of these sates increase implying their more accessibility in the basin volume. After $\epsilon = \epsilon_{PB}$ these two states becomes bi-stable as $E_{3,4}$ lose their stability irrespective of the presence of noise $D \in [0, 0.1]$. From Fig. 3(e) we can see that the BS of fluctuation state around E_0 is equal to 0 before the coupling strength less than ϵ_{IPB}, but it abruptly becomes stable

at $\epsilon = \epsilon_{IPB}$ with BS equal to 1. But there is no change of BS along the $Y-$axis which signifies that no significant influence of noise on it's BS.
